# Associations between twelve common gene polymorphisms and susceptibility to hepatocellular carcinoma: evidence from a meta-analysis

**DOI:** 10.1186/s12957-019-1748-8

**Published:** 2019-12-12

**Authors:** Yi Quan, Jun Yang, Tao Qin, Yufang Hu

**Affiliations:** 1 Department of Clinical Laboratory, Affiliated Hospital of Guilin Medical University, Guilin, Guangxi China; 2 Department of Radiology, Affiliated Hospital of Guilin Medical University, No. 15 of Lequn Road, Guilin, 540001 Guangxi China

**Keywords:** Vitamin D receptor (VDR), Vascular endothelial growth factor (VEGF), Mannose-binding lectin (MBL), Interleukin-18 (*IL-18*), Hepatocellular carcinoma (HCC), Meta-analysis

## Abstract

**Background:**

Associations between polymorphisms in vitamin D receptor (VDR)/vascular endothelial growth factor (VEGF)/interleukin-18 (IL-18)/mannose-binding lectin (MBL) and susceptibility to hepatocellular carcinoma (HCC) were already explored by many studies, yet the results of these studies were inconsistent. The aim of this meta-analysis was to better clarify associations between polymorphisms in *VDR/VEGF/IL-18/MBL* and HCC by combing the results of all relevant studies.

**Methods:**

Eligible publications were searched from PubMed, Embase, WOS, and CNKI. We used Review Manager to combine the results of individual studies.

**Results:**

Thirty studies were included in this study. Combined results revealed that *VDR* rs7975232, *VDR* rs2228570, *VEGF* rs699947, *VEGF* rs3025039, *IL-18* rs1946518, and *MBL* rs7096206 polymorphisms were all significantly associated with HCC in the overall pooled population. We also obtained similar significant associations for *VDR* rs7975232, *VDR* rs2228570, *IL-18* rs1946518, and *MBL* rs7096206 polymorphisms in Asians.

**Conclusions:**

Collectively, this meta-analysis proved that *VDR* rs7975232, *VDR* rs2228570, *VEGF* rs699947, *VEGF* rs3025039, *IL-18* rs1946518, and *MBL* rs7096206 polymorphisms may confer susceptibility to HCC in certain populations.

## Background

Hepatocellular carcinoma (HCC) is one of the leading causes of death all over the world [1, 2]. Although we still did not reveal the exact mechanism of its pathogenesis, it was evident that genetic components were essential in the development of HCC. Firstly, the incidences of HCC in different populations were quite different [3, 4], and genetic background was probably one of the reasons behind differences in disease prevalence across different populations. Secondly, numerous susceptible genetic loci of HCC were also identified and validated by existing genetic association studies [5, 6].

Mannose-binding lectin (MBL) and interleukin-18 (IL-18) are crucial modulators of immunological reactions, whereas vitamin D receptor (VDR) and vascular endothelial growth factor (VEGF) are vital for both immune-regulation and angiogenesis [7–10]. So, if a genetic polymorphism could alter the transcription activity of *VDR/VEGF/IL-18/MBL* or the protein structure of VDR/VEGF/IL-18/MBL, there is a possibility that this polymorphism may lead to the development of chronic inflammatory cellular injuries and also confer susceptibility to many types of malignancy including HCC.

In the past 20 years, many studies explored associations between polymorphisms in *VDR/VEGF/IL-18/MBL* and HCC, yet the conclusions of these studies were somehow inconsistent [11–40]. To better clarify associations between polymorphisms in *VDR/VEGF/IL-18/MBL* and HCC, we designed this study to get a more credible conclusion by combing the results of all relevant studies.

## Methods

We wrote this meta-analysis in accordance with the requirements of the PRISMA guideline [41].

### Literature search and inclusion criteria

To retrieve eligible articles, we searched PubMed, WOS, Embase, and CNKI with keywords listed below: (“vitamin D receptor” or “VDR” or “vascular endothelial growth factor” or “VEGF” or “interleukin 18” or “IL 18” or “mannose-binding lectin” or “Mannose-binding protein” or “MBL” or “MBP”) and (“polymorphism” or “variant” or “variation” or “mutation” or “SNP” or “genome-wide association study” or “genetic association study” or “genotype” or “allele”) and (“hepatocellular carcinoma” or “HCC”). The references of retrieved articles were also screened by us to identify other potentially relevant articles.

To be included in this meta-analysis, some criteria must be met: (I) about associations between polymorphisms in *VDR/VEGF/IL-18/MBL* and HCC in humans; (II) Offer genotypic distribution of *VDR/VEGF/IL-18/MBL* polymorphisms in patients with HCC and controls; (III) full manuscript in English or Chinese is retrievable. Publications were deemed to be ineligible for inclusion if (I) not about polymorphisms in *VDR/VEGF/IL-18/MBL* and HCC; (II) narrative reviews, systematic reviews, or comments; (III) studies only involved HCC patients. We only included the most up to date study for analyses if duplicate publications were found during the literature search.

### Data extraction and quality assessment

Two authors extracted the following essential information from eligible studies: (I) name of the leading author; (II) published year; (III) country of the leading author; (IV) ethnicity of involved participants; (V) number of patients with HCC and controls in each study; (VI) genotype distributions of polymorphisms in *VDR/VEGF/IL-18/MBL* among patients with HCC and controls. *P* values of Hardy-Weinberg equilibrium (HWE) were also calculated.

The authors used the Newcastle-Ottawa scale (NOS) to assess the quality of eligible publications [42]. The score range of NOS is between 0 and 9, when a study got a score of 7 or more, we considered that the methodology quality of this study was good

Two authors extracted data and assessed the quality of eligible studies. The authors wrote to the leadings authors for additional information if essential information was found to be incomplete.

### Statistical analyses

We used Review Manager to combine the results of individual studies. *Z* test was employed to assess associations between polymorphisms in *VDR/VEGF/IL-18/MBL* and susceptibility to HCC. The statistical significance threshold of *P* value was set at 0.05. We used *I*^2^ statistics to assess between-study heterogeneities. We used Random-effect models (DerSimonian-Laird method) to combine the results if *I*^2^ is larger than 50%. Otherwise, fixed-effect models (Mantel-Haenszel method) were used to combine the results [43, 44]. We further carried out subgroup analyses by ethnicity to get ethnic-specific results. We examined the stability of combined results by deleting one study each time and combining the results of the remaining studies. We used funnel plots to estimate whether our combined results may be influenced by publication biases.

## Results

### Characteristics of included studies

**Fig. 1.**
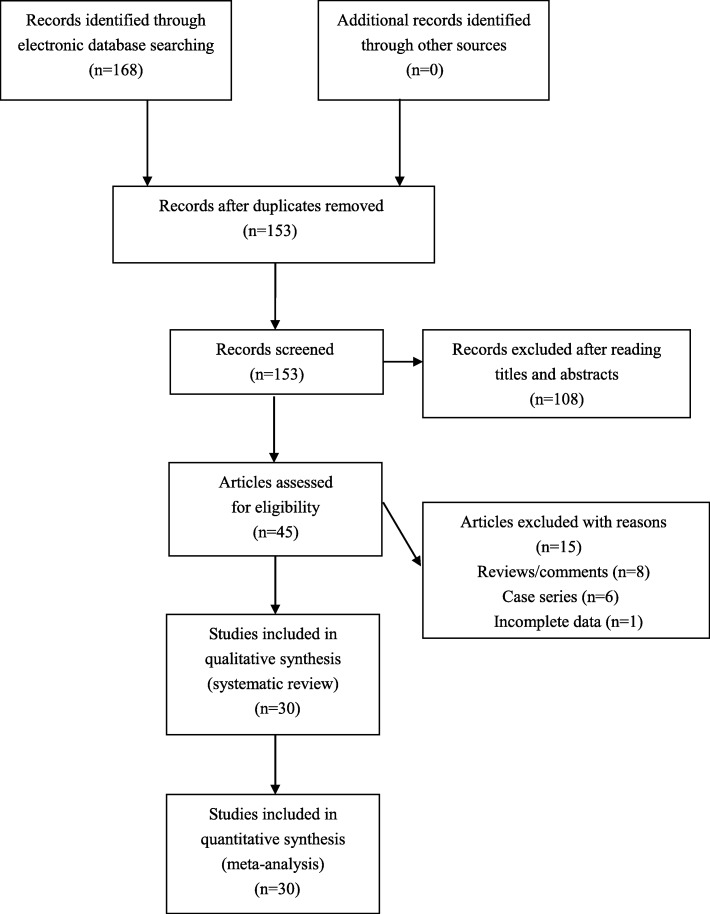
Flowchart of study selection for the present study

We found 168 articles during literature searching. Forty-five articles were assessed for eligibility after excluding unrelated or duplicate articles. We further excluded eight reviews and six case series, and another one publication was excluded because of missing crucial data. Totally, 30 articles were ultimately found to be eligible for inclusion (Fig. 1). Extracted data of eligible articles were summarized in Table 1.

**Table 1:** The characteristics of included studies for this meta-analysis

First author, year	Country	Ethnicity	Type of disease	Medical history of patients	Sample size Case/control	Genotype distribution (wtwt/wtmt/mtmt)		*P* value for HWE	NOS score
						Cases controls			
VDR rs7975232									
Barooah 2019 [11]	India	South Asian	HCC	NA	60/102	49/11/0	59/35/8	0.391	8
Falleti 2010 [12]	Italy	Caucasian	HCC	Viral hepatitis 87%	80/160	27/38/15	53/85/22	0.189	8
Hung 2014 [13]	Taiwan	East Asian	HCC	NA	92/100	65/24/3	55/40/5	0.505	8
Yao 2013 [16]	China	East Asian	HCC	HBV 100%, alcohol intake 34.9%	436/532	112/216/108	114/275/143	0.395	8
VDR rs1544410									
Barooah 2019 [11]	India	South Asian	HCC	NA	60/102	52/8/0	80/16/6	< 0.001	8
Falleti 2010 [12]	Italy	Caucasian	HCC	Viral hepatitis 87%	80/160	33/35/12	45/87/28	0.206	8
Hung 2014 [13]	Taiwan	East Asian	HCC	NA	92/100	85/7/0	89/11/0	0.560	8
Yao 2013 [16]	China	East Asian	HCC	HBV 100%, alcohol intake 34.9%	436/532	112/217/107	142/259/131	0.550	8
VDR rs2228570									
Falleti 2010 [12]	Italy	Caucasian	HCC	Viral hepatitis 87%	80/160	36/36/8	69/73/18	0.843	8
Liu 2015 [14]	China	East Asian	HCC	NA	105/100	41/44/20	23/48/29	0.715	8
Peng 2014 [15]	China	East Asian	HCC	HBV 100%, alcohol intake 90.2%	184/296	54/90/40	77/152/67	0.628	8
Yao 2013 [16]	China	East Asian	HCC	HBV 100%, alcohol intake 34.9%	436/532	131/198/107	102/241/189	0.111	8
VDR rs731236									
Barooah 2019 [11]	India	South Asian	HCC	NA	60/102	48/8/4	71/21/10	<0.001	8
Falleti 2010 [12]	Italy	Caucasian	HCC	Viral hepatitis 87%	80/160	32/38/10	44/88/28	0.160	8
Hung 2014 [13]	Taiwan	East Asian	HCC	NA	92/100	86/6/0	86/14/0	0.452	8
Yao 2013 [16]	China	East Asian	HCC	HBV 100%, alcohol intake 34.9%	436/532	115/212/109	137/252/143	0.226	8
VEGF rs699947									
Liu 2017 [19]	China	East Asian	HCC	HBV 60.2%, alcohol intake 60.8%	476/526	301/157/18	290/202/34	0.882	8
Machado 2014 [20]	Portugal	Caucasian	HCC	Alcohol intake 100%	26/101	7/14/5	19/49/33	0.914	7
Ratnasari 2017 [22]	Indonesia	East Asian	HCC	HBV58%, HCV 11%	44/59	18/21/5	23/30/6	0.402	7
Wu 2009 [23]	China	East Asian	HCC	NA	92/90	48/40/4	58/28/4	0.792	8
Wu 2013 [24]	China	East Asian	HCC	HBV48.5%	101/110	79/21/1	91/17/2	0.271	8
VEGF rs1570360									
Baitello 2016 [17]	Canada	Mixed	HCC	HBV 50%, HCV 21%, alcohol intake 56%	102/127	61/35/6	73/47/7	0.875	8
Wu 2009 [23]	China	East Asian	HCC	NA	90/99	66/24/0	72/27/0	0.116	8
Wu 2013 [24]	China	East Asian	HCC	HBV48.5%	101/110	83/17/1	75/31/4	0.723	8
VEGF rs2010963									
Liu 2017 [19]	China	East Asian	HCC	HBV 60.2%, alcohol intake 60.8%	476/526	162/232/82	200/248/78	0.937	8
Ratnasari 2016 [21]	Indonesia	East Asian	HCC	HBV56.5%, HCV 10.8%	46/136	16/29/1	26/105/5	<0.001	7
Wu 2009 [23]	China	East Asian	HCC	NA	92/99	34/40/18	34/52/13	0.320	8
Wu 2013 [24]	China	East Asian	HCC	HBV48.5%	101/110	28/52/21	35/51/24	0.506	8
VEGF rs3025039									
Baitello 2016 [17]	Canada	Mixed	HCC	HBV 50%, HCV 21%, alcohol intake 56%	102/127	72/30/0	90/37/0	0.055	8
Giacalone 2011 [18]	Italy	Caucasian	HCC	NA	96/162	81/14/1	120/38/4	0.636	8
Liu 2017 [19]	China	East Asian	HCC	HBV 60.2%, alcohol intake 60.8%	476/526	359/112/5	370/140/16	0.536	8
Wu 2009 [23]	China	East Asian	HCC	NA	92/99	63/26/3	68/30/1	0.239	8
Yvamoto 2015 [25]	Brazil	Mixed	HCC	Alcohol intake 47.1%	228/56	164/64/0	43/13/0	0.326	7
IL-18 rs187238									
Bakr 2018 [26]	Egypt	South Asian	HCC	HCV 100%	90/90	66/22/2	33/65/1	<0.001	8
Bao 2015 [27]	China	East Asian	HCC	HBV 100%	153/165	122/28/3	106/54/5	0.548	8
Chen 2012 [28]	China	East Asian	HCC	NA	228/300	159/59/10	173/115/12	0.183	7
Dai 2017 [29]	China	East Asian	HCC	HBV 100%, alcohol intake 42%	245/250	187/49/9	183/65/2	0.142	8
Karra 2015 [30]	India	South Asian	HCC	HBV 100%	271/280	123/134/14	159/108/13	0.320	7
Kim 2009 [31]	Korea	East Asian	HCC	HBV 100%	56/558	37/17/2	434/122/2	0.031	7
Lau 2016 [32]	Taiwan	East Asian	HCC	Alcohol intake 63.5%	342/559	266/73/3	476/78/5	0.370	8
Migita 2009 [33]	Japan	East Asian	HCC	HBV 100%	47/63	43/3/1	52/10/1	0.531	7
Teixeira 2009 [34]	Brazil	Mixed	HCC	Viral hepatitis 67.8%, alcohol intake 63.4%	112/202	57/48/7	100/84/18	0.952	7
Zhang 2016 [35]	China	East Asian	HCC	HBV 100%	109/127	82/25/2	99/24/4	0.110	8
IL18 rs1946518									
Bakr 2018 [26]	Egypt	South Asian	HCC	HCV 100%	90/99	13/34/43	17/45/37	0.603	8
Bao 2015 [27]	China	East Asian	HCC	HBV 100%	153/165	37/73/43	41/76/48	0.322	8
Chen 2012 [28]	China	East Asian	HCC	NA	228/300	47/126/55	83/156/61	0.429	7
Dai 2017 [29]	China	East Asian	HCC	HBV 100%, alcohol intake 42%	247/250	62/118/67	64/124/62	0.900	8
Karra 2015 [30]	India	South Asian	HCC	HBV 100%	271/280	70/152/49	102/144/34	0.119	7
Lau 2016 [32]	Taiwan	East Asian	HCC	Alcohol intake 63.5%	342/559	88/167/87	148/276/135	0.777	8
Migita 2009 [33]	Japan	East Asian	HCC	HBV 100%	47/63	13/26/8	20/30/13	0.777	7
Teixeira 2009 [34]	Brazil	Mixed	HCC	Viral hepatitis 67.8%, alcohol intake 63.4%	112/202	38/56/18	85/105/12	0.202	7
Zhang 2016 [35]	China	East Asian	HCC	HBV 100%	109/127	22/55/32	38/66/23	0.127	8
MBL rs7096206									
Eurich 2011 [36]	Germany	Caucasian	HCC	NA	62/115	27/34/1	76/37/2	0.292	7
Gu 2016 [37]	China	East Asian	HCC	NA	334/171	232/95/7	131/33/7	0.015	8
Lin 2015 [38]	China	East Asian	HCC	Alcohol intake 77.7%	220/220	125/86/9	153/65/2	0.082	8
Su 2016 [40]	China	East Asian	HCC	HBV 70.2%	315/315	207/91/17	239/72/4	0.583	8
MBL rs1800450				NA					
Gu 2016 [37]	China	East Asian	HCC	NA	334/171	234/89/11	104/59/8	0.920	8
Segat 2008 [39]	Italy	Caucasian	HCC	NA	215/164	127/78/10	102/49/13	0.050	7
Su 2016 [40]	China	East Asian	HCC	HBV 70.2%	308/315	208/88/20	239/69/7	0.450	8

Abbreviations: *HWE* Hardy-Weinberg equilibrium, *NOS* Newcastle-Ottawa scale, *NA* not available, *HBV* hepatitis B virus infection, *HCV* hepatitis C virus infection

### Meta-analyses results for polymorphisms in VDR and HCC

Six studies were eligible for estimation of associations between polymorphisms in *VDR* and HCC. *VDR* rs7975232 (dominant comparison OR = 1.58, 95% CI 1.04–2.39; over-dominant comparison OR = 0.80, 95% CI 0.65–0.98) and rs2228570 (dominant comparison OR = 1.54, 95% CI 1.25–1.89; recessive comparison OR = 0.67, 95 % CI 0.54–0.84; allele comparison OR = 1.34, 95% CI 1.06–1.68) polymorphisms were found to be significantly associated with HCC in overall combined analyses. Subgroup analyses showed similar positive findings for rs7975232 (dominant comparison) and rs2228570 (dominant, recessive, and allele comparisons) polymorphisms in East Asians (see Table 2 and Additional file 1: Supplementary Figure S1).

**Table 2:** Meta-analyses results of the current study

Variables	Sample size	Dominant comparison		Recessive comparison		Over-dominant comparison		Allele comparison	
		*P* value OR (95%CI) *I*^2^ statistic		*P* value	OR (95%CI) *I*^*2*^ statistic	*P* value OR (95% CI) *I*^2^ statistic		*P* value	OR (95%CI) *I*^2^ statistic
*VDR* rs7975232									
Overall	668/894	*0.03* 60%	*1.58 (1.04–2.39)*	0.42 31%	0.90 (0.69–1.17)	*0.03* 44%	*0.80 (0.65–0.98)*	0.09 76%	1.41 (0.94–2.12)
East Asian	528/632	*0.02* 40%	*1.39 (1.06–1.81)*	0.40 0%	0.88 (0.67–1.17)	0.28 62%	0.75 (0.45–1.26)	0.17 55%	1.30 (0.89–1.89)
VDR rs1544410									
Overall	668/894	0.26 44%	1.15 (0.90–1.45)	0.62 8%	0.93 (0.71–1.22)	0.54 0%	0.93 (0.75–1.16)	0.30 50%	1.09 (0.93–1.27)
East Asian	528/632	0.98 0%	1.00 (0.74–1.34)	0.91 0%	0.98 (0.75–1.30)	0.90 0%	1.02 (0.79–1.30)	0.96 0%	1.00 (0.83–1.19)
VDR rs2228570									
Overall	805/1088	*< 0.0001* 46%	*1.54 (1.25–1.89)*	*0.0004* 19%	*0.67 (0.54–0.84)*	0.58 0%	0.95 (0.79–1.14)	*0.01* 59%	*1.34 (1.06–1.68)*
East Asian	725/928	*< 0.0001* 45%	*1.63 (1.31–2.04)*	*0.0003* 40%	*0.66 (0.53–0.83)*	0.58 0%	0.95 (0.78–1.15)	*0.01* 65%	*1.40 (1.08–1.82)*
VDR rs731236									
Overall	668/894	0.06 43%	1.25 (0.99–1.58)	0.26 0%	0.86 (0.66–1.12)	0.42 38%	0.92 (0.74–1.14)	0.06 42%	1.16 (0.99–1.36)
East Asian	528/632	0.44 57%	1.34 (0.64–2.82)	0.51 0%	0.91 (0.68–1.21)	0.54 66%	0.77 (0.33–1.78)	0.39 55%	1.08 (0.91–1.29)
VEGF rs699947									
Overall	739/886	0.92 54%	1.02 (0.69–1.52)	*0.04* 0%	*0.63 (0.41–0.98)*	0.61 45%	0.95 (0.77–1.17)	0.61 51%	1.08 (0.80–1.46)
East Asian	713/785	0.84 64%	1.05 (0.66–1.66)	0.10 0%	0.67 (0.41–1.08)	0.70 56%	1.08 (0.72–1.65)	0.99 59%	1.00 (0.70–1.42)
VEGF rs1570360									
Overall	293/336	0.12 37%	1.31 (0.93–1.85)	0.57 19%	0.75 (0.29–1.98)	0.17 7%	0.78 (0.55–1.11)	0.13 49%	1.26 (0.94–1.70)
East Asian	191/209	0.28 60%	1.49 (0.72–3.06)	0.24 0%	0.27 (0.03–2.41)	0.15 44%	0.71 (0.45–1.13)	0.28 64%	1.46 (0.73–2.91)
VEGF rs2010963									
Overall	715/871	0.79 55%	1.05 (0.72–1.54)	0.26 0%	1.17 (0.89–1.55)	0.80 48%	0.97 (0.80–1.19)	0.32 13%	0.93 (0.81–1.07)
East Asian	715/871	0.79 55%	1.05 (0.72–1.54)	0.26 0%	1.17 (0.89–1.55)	0.80 48%	0.97 (0.80–1.19)	0.32 13%	0.93 (0.81–1.07)
VEGF rs3025039									
Overall	994/970	0.08 12%	1.20 (0.98–1.48)	0.08 38%	0.50 (0.23–1.09)	0.21 0%	0.87 (0.71–1.08)	*0.05* 28%	*1.21 (1.00–1.46)*
East Asian	568/625	0.10 0%	1.24 (0.96–1.59)	0.87 69%	0.83 (0.09–7.41)	0.25 0%	0.86 (0.66–1.11)	0.06 34%	1.24 (0.99–1.56)
IL-18 rs187238									
Overall	1653/2594	0.38 85%	1.19 (0.81–1.77)	0.50 16%	1.14 (0.78–1.66)	0.26 88%	0.77 (0.49–1.21)	0.56 78%	1.09 (0.82–1.43)
East Asian	1180/2022	0.62 81%	1.11 (0.73–1.70)	0.27 33%	1.33 (0.80–2.22)	0.49 81%	0.86 (0.55–1.34)	0.76 78%	1.06 (0.74–1.50)
South Asian	361/370	0.60 97%	1.70 (0.24–12.29)	0.65 0%	1.19 (0.57–2.47)	0.53 98%	0.45 (0.04–5.35)	0.69 92%	1.25 (0.42–3.66)
HBV	881/1443	0.90 78%	1.03 (0.65–1.63)	0.23 43%	1.38 (0.81–2.33)	0.73 78%	0.92 (0.57–1.48)	0.96 74%	1.01 (0.70–1.46)
IL18 rs1946518									
Overall	1599/2045	*0.002* 0%	*0.79 (0.68–0.92)*	*0.004* 30%	*1.26 (1.08–1.48)*	0.75 0%	1.02 (0.90–1.17)	*0.002* 59%	*0.78 (0.67–0.91)*
East Asian	1126/1464	0.09 0%	0.86 (0.71–1.02)	0.15 0%	1.14 (0.95–1.37)	0.79 0%	1.02 (0.87–1.19)	*0.04* 68%	*0.80 (0.65–0.99)*
South Asian	589/679	*0.001* 0%	*0.66 (0.51–0.85)*	*0.02* 0%	*1.57 (1.09–2.27)*	0.98 54%	0.99 (0.61–1.61)	*0.002* 0%	*0.72 (0.59–0.89)*
HBV	827/885	*0.01* 9%	*0.77 (0.62–0.95*	0.06 21%	1.25 (0.99–1.57)	0.52 0%	1.06 (0.88–1.29)	*0.03* 73%	*0.73 (0.55–0.96)*
MBL rs7096206									
Overall	931/821	*< 0.0001 0.59 (0.48–0.73)* 0%		0.37 70%	1.81 (0.50–6.59)	*< 0.0001* 0%	*1.59 (1.28–1.97)*	*< 0.0001* 0%	*0.63 (0.53–0.76)*
East Asian	869/706	*< 0.0001* 0%	*0.62 (0.50–0.78)*	0.35 79%	2.08 (0.44–9.80)	*0.0005* 0%	*1.50 (1.19–1.88)*	*< 0.0001* 4%	*0.65 (0.53–0.79)*
MBL rs1800450									
Overall	857/650	0.85 79%	0.95 (0.58–1.55)	0.91 77%	1.06 (0.37–3.06)	0.70 75%	1.10 (0.69–1.74)	0.95 80%	0.99 (0.65–1.50)
East Asian	642/486	0.99 90%	0.99 (0.44–2.23)	0.61 81%	1.47 (0.34–6.30)	0.99 86%	1.00 (0.49–2.03)	0.95 90%	0.98 (0.48–1.99)

Abbreviations: *OR* odds ratio, *CI* confidence interval, *NA* not available, *HBV* hepatitis B virus infection

The values in italics represent that there is statistically significant differences between cases and controls

### Meta-analyses results for polymorphisms in VEGF and HCC

Nine studies were eligible for the estimation of associations between polymorphisms in *VEGF* and HCC. *VEGF* rs699947 (recessive comparison OR = 0.63, 95% CI 0.41–0.98) and rs3025039 (allele comparison OR = 1.21, 95% CI 1.00–1.46) polymorphisms were found to be significantly associated with HCC in overall combined analyses. Nevertheless, we did not observe any positive associations in subgroup analyses (see Table 2 and Additional file 1: Supplementary Figure S1).

### Meta-analyses results for polymorphisms in IL-18 and HCC

Ten studies were eligible for the estimation of associations between polymorphisms in *IL-18* and HCC. *IL-18* rs1946518 (dominant comparison OR = 0.79, 95% CI 0.68–0.92; recessive comparison OR = 1.26, 95 % CI 1.08–1.48; allele comparison OR = 0.78, 95% CI 0.67–0.91) polymorphism was found to be significantly associated with HCC in overall combined analyses. Subgroup analyses showed similar positive findings for rs1946518 polymorphism in East Asians (allele comparison), South Asians (dominant, recessive, and allele comparisons), and those with hepatitis B virus (HBV) infection (dominant and allele comparisons) (see Table 2 and Additional file 1: Supplementary Figure S1).

### Meta-analyses results for polymorphisms in MBL and HCC

Five studies were eligible for the estimation of associations between polymorphisms in *MBL* and HCC. *MBL* rs7096206 (dominant comparison OR = 0.59, 95% CI 0.48–0.73; over-dominant comparison OR = 1.59, 95% CI 1.28–1.97; allele comparison: OR = 0.63, 95% CI 00.53–0.76) polymorphism was found to be significantly associated with HCC in overall combined analyses. Subgroup analyses showed similar positive findings for rs7096206 polymorphism in East Asians (dominant, over-dominant, and allele comparisons) (see Table 2 and Additional file 1: Supplementary Figure S1).

### Sensitivity analyses

We examined the stability of combined results by deleting one study each time and combining the results of the remaining studies. The trends of associations remained consistent in sensitivity analyses, which indicated that the combined results were statistically stable.

### Publication biases

Funnels plots were employed to estimate whether our combined results may be influenced by publication biases. Funnel plots of every comparison were symmetrical, which indicated that the combined results were unlikely to be seriously impacted by overt publication biases.

## Discussion

The combined results of this meta-analysis revealed that *VDR* rs7975232, *VDR* rs2228570, *VEGF* rs699947, *VEGF* rs3025039, *IL-18* rs1946518, and *MBL* rs7096206 polymorphisms were significantly associated with susceptibility to HCC in certain populations. The trends of associations remained consistent in sensitivity analyses, which indicated that the combined results were statistically stable.

To better understand the combined results of this meta-analysis, some points should be considered. First, past basic studies revealed that all investigated polymorphisms were either correlated with altered transcription activity or protein structure [45–48]. So, these variations may influence the biological function of *VDR/VEGF/IL-18/MBL*, result in immune dysfunction, cause chronic inflammatory hepatocellular injury, and ultimately confer susceptibility to HCC. Thus, our meta-analysis may be statistically insufficient to observe the real underlying associations between polymorphisms in *VDR/VEGF/IL-18/MBL* and HCC in certain subgroups. Therefore, future studies still need to confirm our findings. Second, we noticed that most eligible studies were from Asian countries, whereas studies in other countries were highly scarce, so scholars from European and African countries should also try to examine associations between polymorphisms in *VDR/VEGF/IL-18/MBL* and HCC. Besides, considering the functional importance of VDR/VEGF/IL-18/MBL in regulating inflammatory reactions and angiogenesis, future studies also need to test the relationship between polymorphisms in *VDR/VEGF/IL-18/MBL* and other types of malignancies. Third, the etiology of HCC is very complicated, so we highly recommend further genetic association studies to explore the effects of haplotypes and gene-gene interactions on disease susceptibility [49]. Fourth, we aimed to investigate associations between all polymorphisms in *VDR/VEGF/IL-18/MBL* and HCC in the very beginning. However, we did not find any study on other *VDR/VEGF/IL-18/MBL* polymorphisms, so we only focused on 12 polymorphisms in this meta-analysis. Fifth, it is worth noting that Zhu et al. [50] also performed a meta-analysis about *IL-18* polymorphisms and HCC in 2016. Based on combined analyses of eight eligible studies with 3572 subjects, they did not find any positive results regarding *IL-18* polymorphisms and HCC in general or subgroup analyses. Since our pooled analyses about *IL-18* polymorphisms were based on more eligible studies and larger sample sizes, our results should be more statistically robust. Nevertheless, studies with larger sample sizes are still warranted to test the genetic associations between *IL-18* polymorphisms and HCC in the future.

Some limitations of this meta-analysis should also be mentioned. Firstly, the results regarding associations between polymorphisms in *VDR/VEGF/IL-18/MBL* and HCC were based on combining unadjusted findings of eligible studies due to the lack of raw data [51]. Secondly, the relationship between polymorphisms in *VDR/VEGF/IL-18/MBL* and HCC may also be affected by environmental factors. Unfortunately, the majority of eligible studies only focused on associations between polymorphisms in *VDR/VEGF/IL-18/MBL* and HCC, so we could not explore genetic-environmental interactions in this meta-analysis [52]. Thirdly, grey literatures were not searched. So although funnel plots of every comparison were symmetrical, it is still possible that the combined results may be affected by publication biases [53].

## Conclusion

In summary, this meta-analysis proved that *VDR* rs7975232, *VDR* rs2228570, *VEGF* rs699947, *VEGF* rs3025039, *IL-18* rs1946518, and *MBL* rs7096206 polymorphisms may confer susceptibility to HCC in certain populations. These results also indicated that VDR, VEGF, IL-18, and MBL may involve in the development of HCC. However, the combined results of this meta-analysis should still be verified by studies with larger sample sizes.

## Supplementary information

**Additional file 1: Figure S1.** Forest plots of investigated polymorphisms.

## Data Availability

The current study was based on the results of relevant published studies.

## Abbreviations

VDR: Vitamin D receptor
VEGF: Vascular endothelial growth factor
MBL: Mannose-binding lectin
IL-18: Interleukin-18
HCC: Hepatocellular carcinoma
HWE: Hardy-Weinberg equilibrium
NOS: Newcastle-Ottawa scale
OR: Odds ratios
CI: Confidence intervals
