# Supplementary material for: Associations between twelve common gene polymorphisms and susceptibility to hepatocellular carcinoma: evidence from a meta-analysis
Source: World J Surg Oncol. 2019 Dec 12;17:216. doi: 10.1186/s12957-019-1748-8 (PMC6909495; doi:10.1186/s12957-019-1748-8)
Supplement: Supplementary file 1 — Additional file 1: Figure S1. Forest plots of investigated polymorphisms. [file 12957_2019_1748_MOESM1_ESM.docx]

Supplementary figure 1. Forest plots of investigated polymorphisms


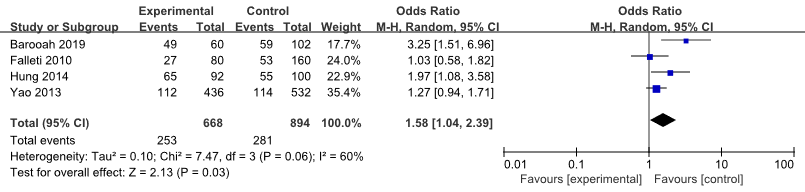


Forest plot of ***VDR* rs7975232** polymorphism and HCC under dominant comparison


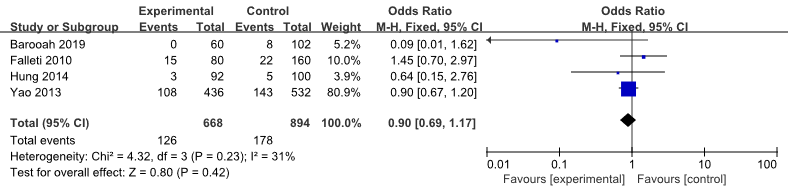


Forest plot of ***VDR* rs7975232** polymorphism and HCC under recessive comparison


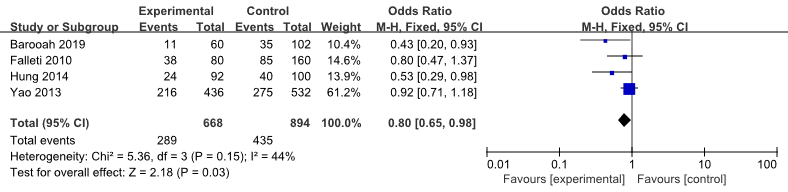


Forest plot of ***VDR* rs7975232** polymorphism and HCC under additive comparison


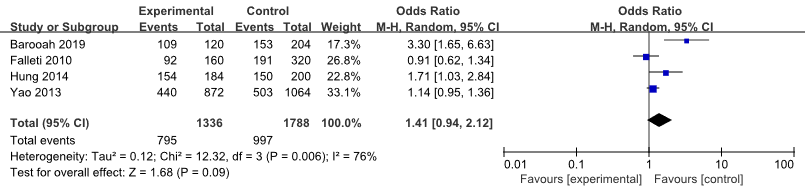


Forest plot of ***VDR* rs7975232** polymorphism and HCC under allele comparison


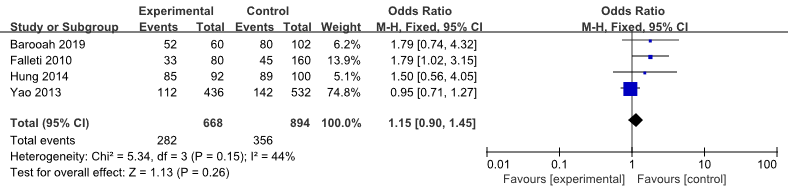


Forest plot of ***VDR* rs1544410** polymorphism and HCC under dominant comparison


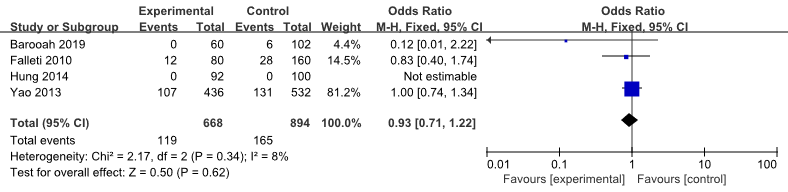


Forest plot of ***VDR* rs1544410** polymorphism and HCC under recessive comparison


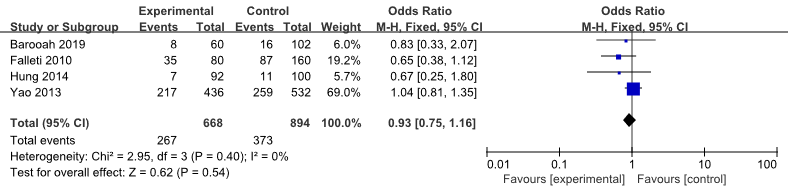


Forest plot of ***VDR* rs1544410** polymorphism and HCC under additive comparison


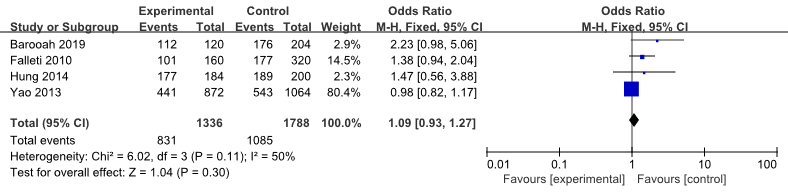


Forest plot of ***VDR* rs1544410** polymorphism and HCC under allele comparison


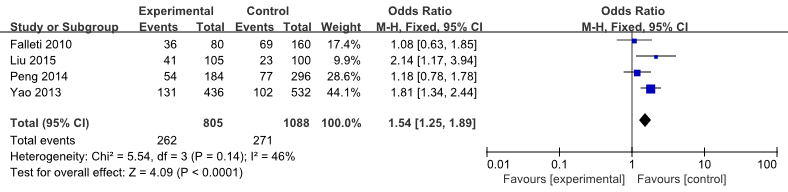


Forest plot of ***VDR* rs2228570** polymorphism and HCC under dominant comparison


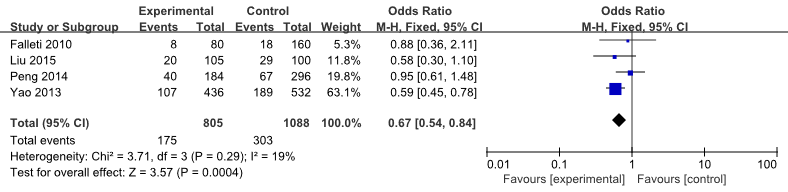


Forest plot of ***VDR* rs2228570** polymorphism and HCC under recessive comparison


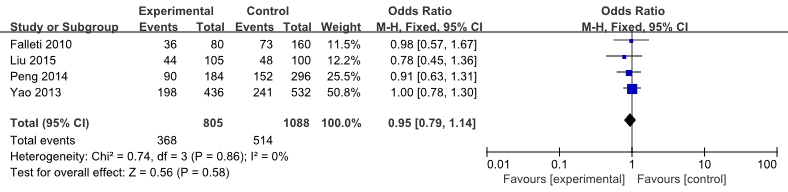


Forest plot of ***VDR* rs2228570** polymorphism and HCC under additive comparison


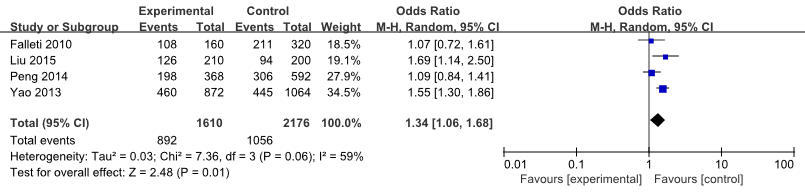


Forest plot of ***VDR* rs2228570** polymorphism and HCC under allele comparison


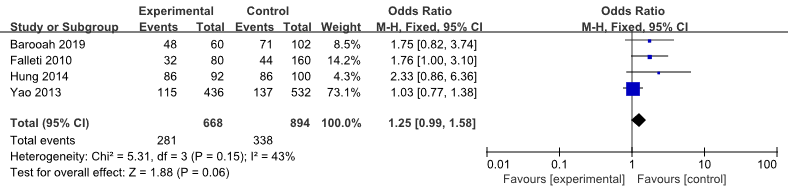


Forest plot of ***VDR* rs731236** polymorphism and HCC under dominant comparison


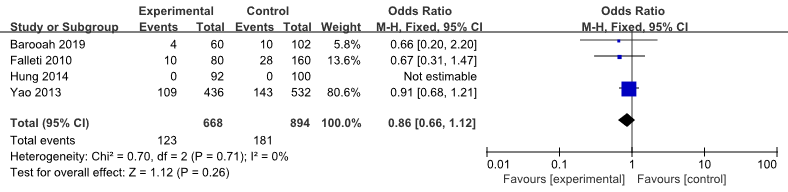


Forest plot of ***VDR* rs731236** polymorphism and HCC under recessive comparison


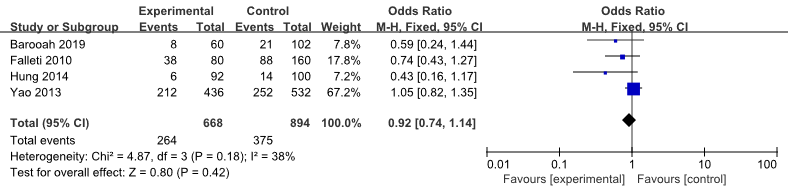


Forest plot of ***VDR* rs731236** polymorphism and HCC under additive comparison


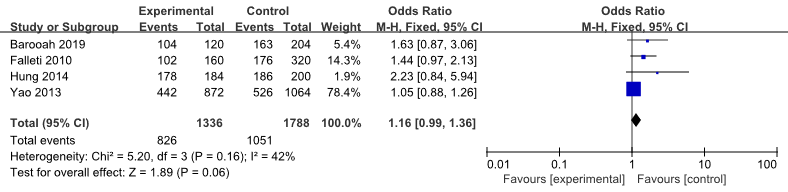


Forest plot of ***VDR* rs731236** polymorphism and HCC under allele comparison


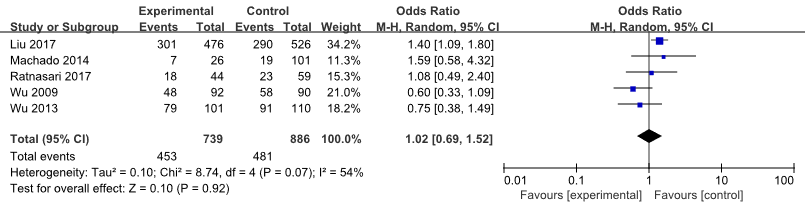


Forest plot of ***VEGF* rs699947** polymorphism and HCC under dominant comparison


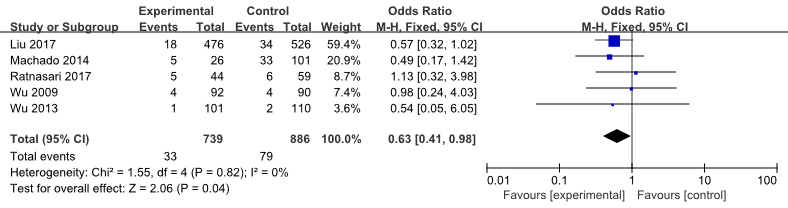


Forest plot of ***VEGF* rs699947** polymorphism and HCC under recessive comparison


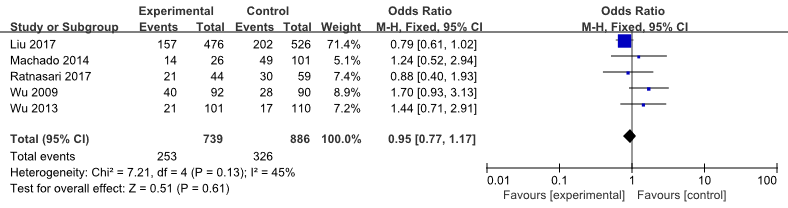


Forest plot of ***VEGF* rs699947** polymorphism and HCC under additive comparison


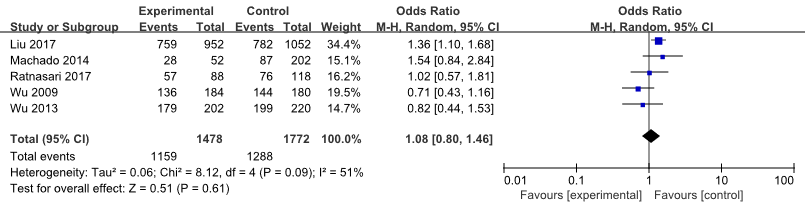


Forest plot of ***VEGF* rs699947** polymorphism and HCC under allele comparison


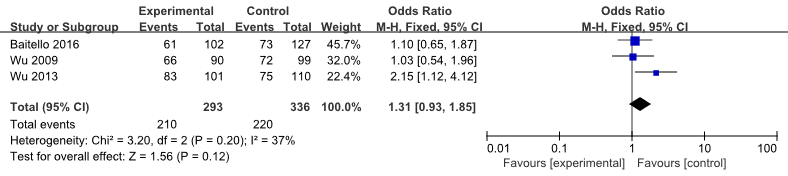


Forest plot of ***VEGF* rs1570360** polymorphism and HCC under dominant comparison


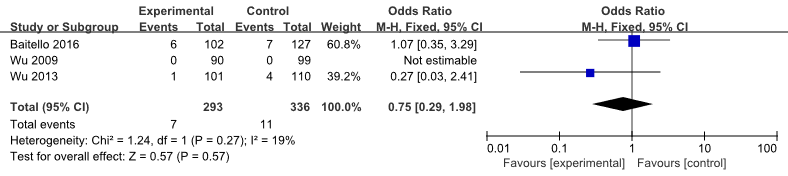


Forest plot of ***VEGF* rs1570360** polymorphism and HCC under recessive comparison


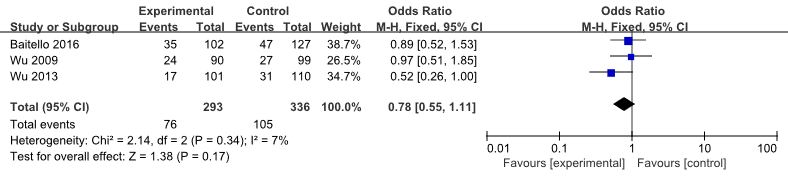


Forest plot of ***VEGF* rs1570360** polymorphism and HCC under additive comparison


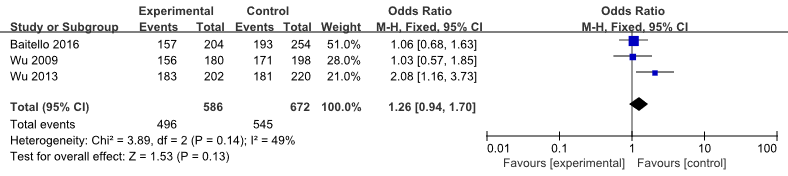


Forest plot of ***VEGF* rs1570360** polymorphism and HCC under allele comparison


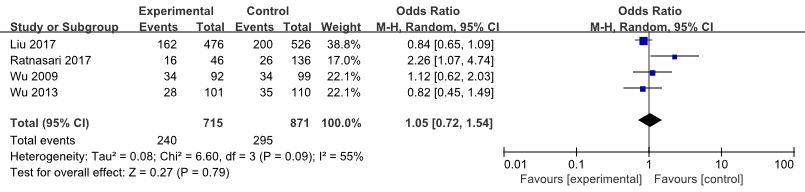


Forest plot of ***VEGF* rs2010963** polymorphism and HCC under dominant comparison


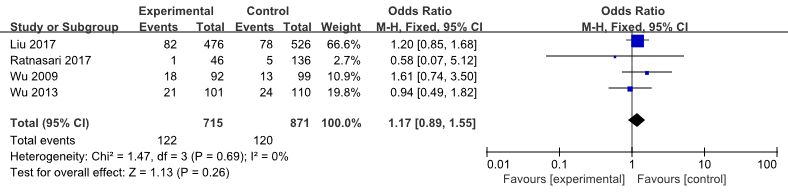


Forest plot of ***VEGF* rs2010963** polymorphism and HCC under recessive comparison


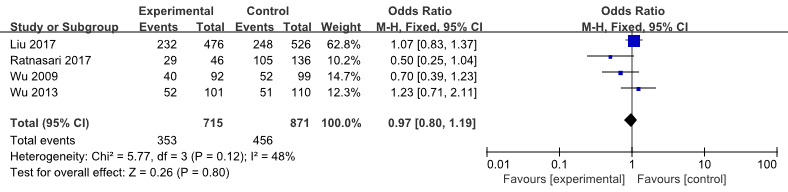


Forest plot of ***VEGF* rs2010963** polymorphism and HCC under additive comparison


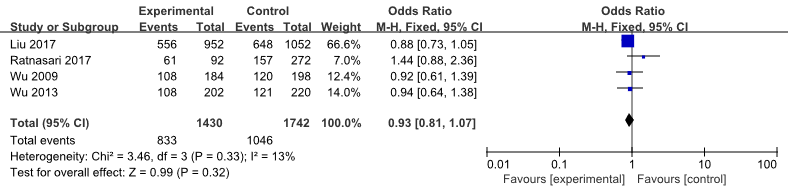


Forest plot of ***VEGF* rs2010963** polymorphism and HCC under allele comparison


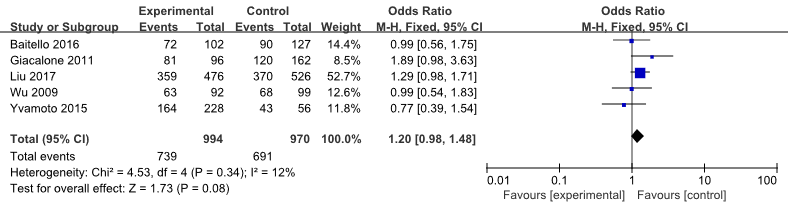


Forest plot of ***VEGF* rs3025039** polymorphism and HCC under dominant comparison


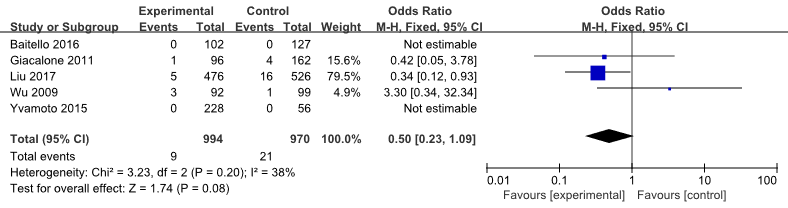


Forest plot of ***VEGF* rs3025039** polymorphism and HCC under recessive comparison


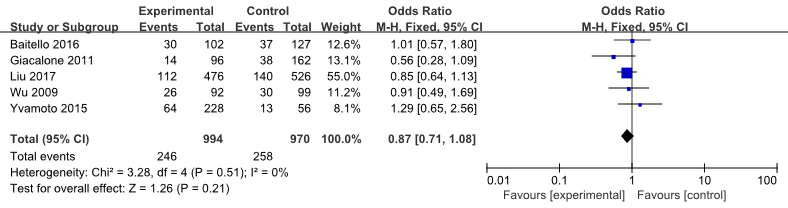


Forest plot of ***VEGF* rs3025039** polymorphism and HCC under additive comparison


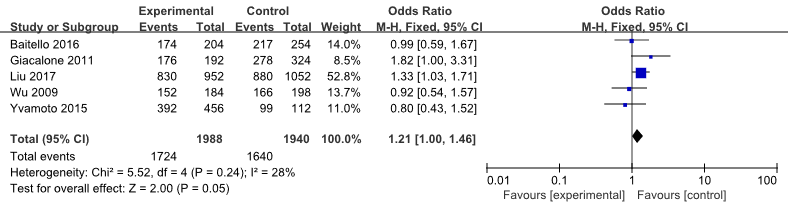


Forest plot of ***VEGF* rs3025039** polymorphism and HCC under allele comparison


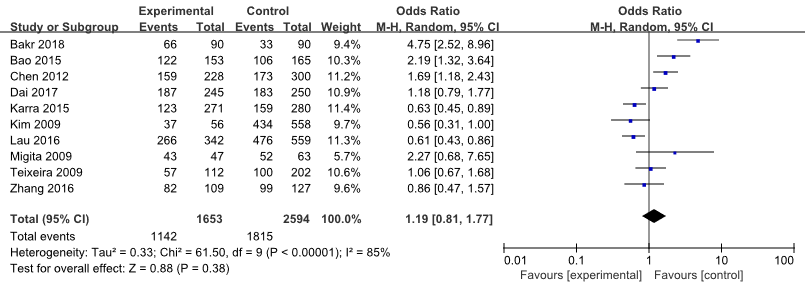


Forest plot of ***IL-18* rs187238** polymorphism and HCC under dominant comparison


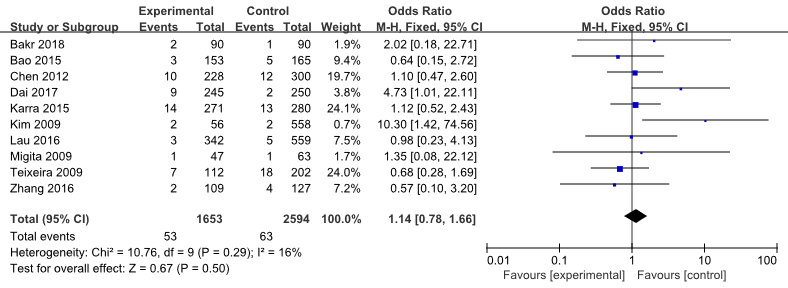


Forest plot of ***IL-18* rs187238** polymorphism and HCC under recessive comparison


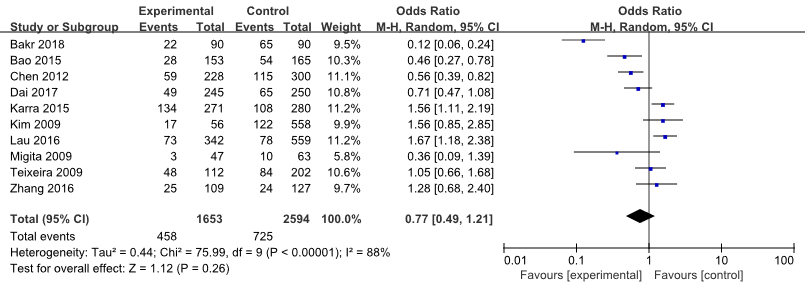


Forest plot of ***IL-18* rs187238** polymorphism and HCC under additive comparison


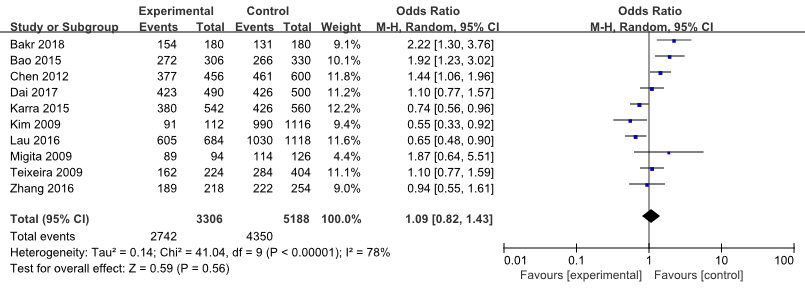


Forest plot of ***IL-18* rs187238** polymorphism and HCC under allele comparison


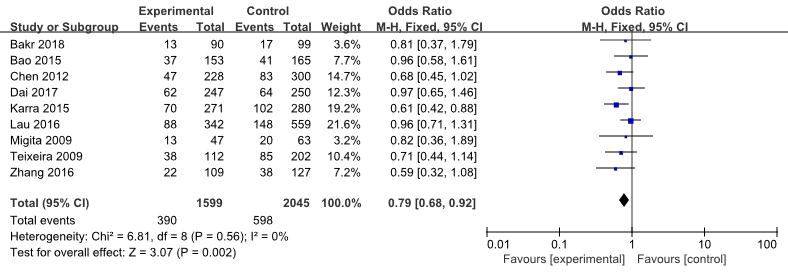


Forest plot of ***IL18* rs1946518** polymorphism and HCC under dominant comparison


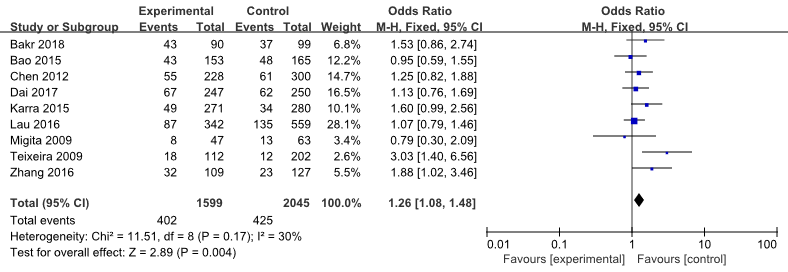


Forest plot of ***IL18* rs1946518** polymorphism and HCC under recessive comparison


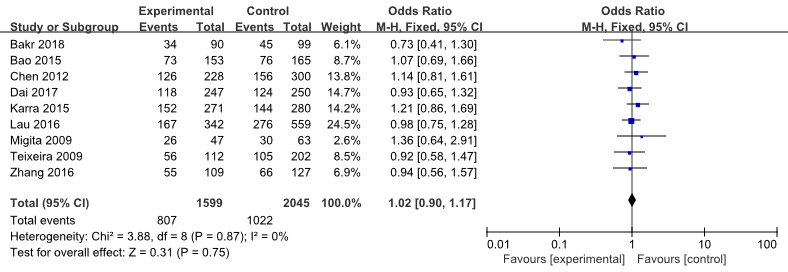


Forest plot of ***IL18* rs1946518** polymorphism and HCC under additive comparison


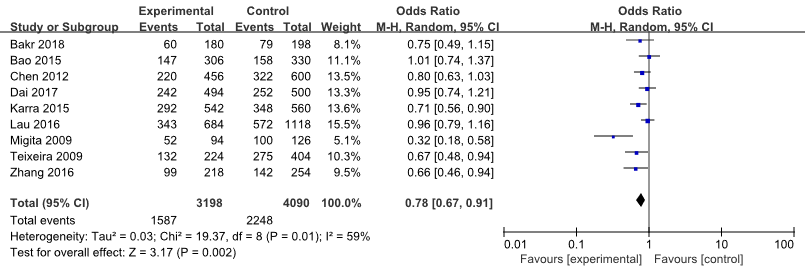


Forest plot of ***IL18* rs1946518** polymorphism and HCC under allele comparison


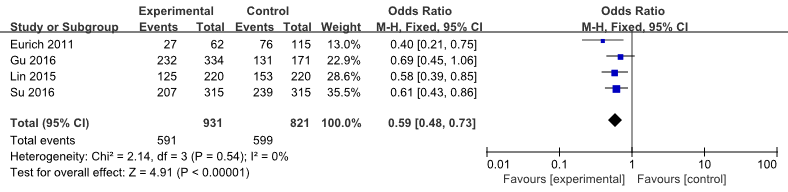


Forest plot of ***MBL* rs7096206** polymorphism and HCC under dominant comparison


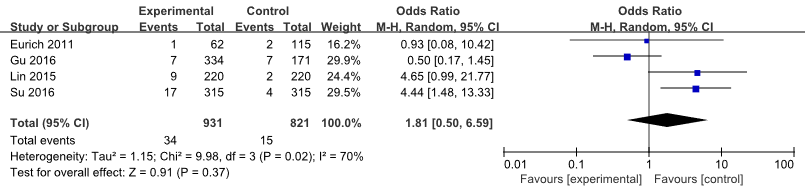


Forest plot of ***MBL* rs7096206** polymorphism and HCC under recessive comparison


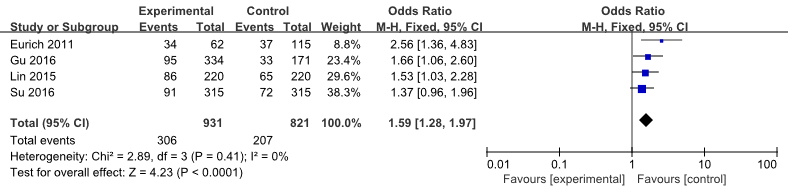


Forest plot of ***MBL* rs7096206** polymorphism and HCC under additive comparison


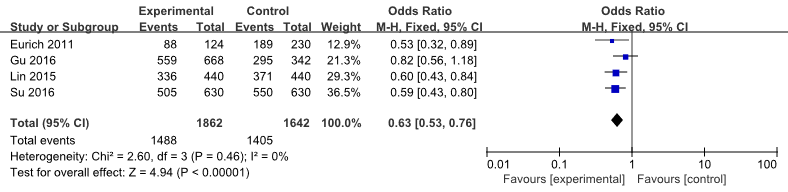


Forest plot of ***MBL* rs7096206** polymorphism and HCC under allele comparison


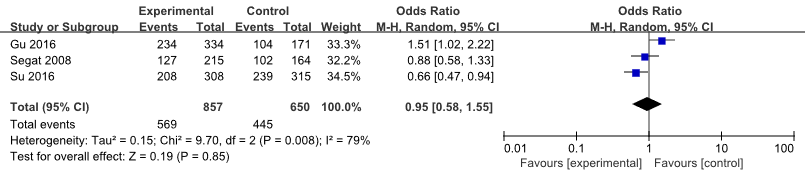


Forest plot of ***MBL* rs1800450** polymorphism and HCC under dominant comparison


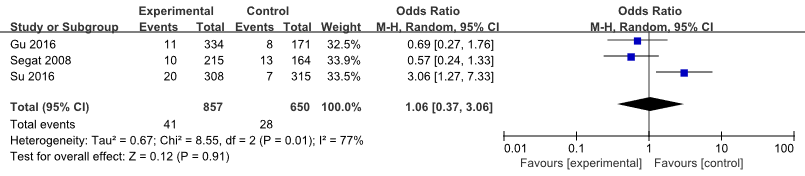


Forest plot of ***MBL* rs1800450** polymorphism and HCC under recessive comparison


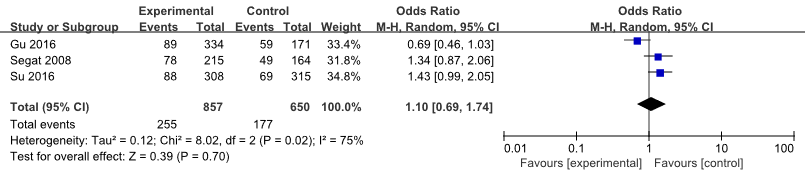


Forest plot of ***MBL* rs1800450** polymorphism and HCC under additive comparison


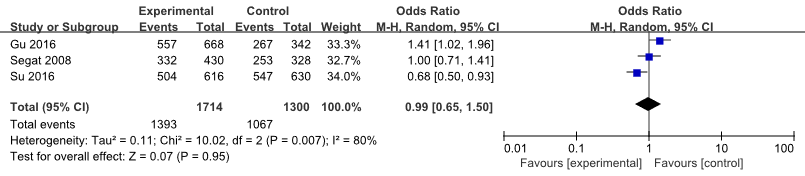


Forest plot of ***MBL* rs1800450** polymorphism and HCC under allele comparison
